# Postoperative chemoradiotherapy with cisplatin is superior to radioimmunotherapy with cetuximab and radiotherapy alone: Analysis of the Austrian head and neck cancer registry of the AGMT

**DOI:** 10.1007/s00508-021-01939-3

**Published:** 2021-09-15

**Authors:** Teresa Magnes, Sandro M. Wagner, Thomas Melchardt, Lukas Weiss, Gabriel Rinnerthaler, Florian Huemer, Michael Kopp, Simon Peter Gampenrieder, Beate Mayrbäurl, Thorsten Füreder, Daniel Lenger, Johannes Andel, Alexander Egle, Richard Greil

**Affiliations:** 1 IIIrd Medical Department, Paracelsus Medical University, Müllner Hauptstraße 48, 5020 Salzburg, Austria; 2 Salzburg Cancer Research Institute, Müllner Hauptstraße 48, 5020 Salzburg, Austria; 3 Abteilung für Innere Medizin IV, Hämatologie, internistische Onkologie und Palliativmedizin, Nephrologie und Dialyse, Klinikum Wels—Grieskirchen GmbH, Grieskirchner Straße 42, 4600 Wels, Austria; 4 Universitätsklinik für Innere Medizin I: Klinische Abteilung für Onkologie, AKH Meduni Wien, Währinger Gürtel 18–20, 1090 Vienna, Austria; 5 Med. Campus III—Hämatologie und Internistische Onkologie, Kepler Universitätsklinikum GmbH Linz, Krankenhausstraße 9, 4021 Linz, Austria; 6 Abteilung für Innere Medizin 2, Pyhrn-Eisenwurzen Klinikum Steyr, Sierninger Straße 170, 4400 Steyr, Austria; 7 Cancer Cluster Salzburg, Müllner Hauptstraße 48, 5020 Salzburg, Austria

**Keywords:** Head and neck cancer, Squamous cell carcinoma of head and neck, Adjuvant, Cisplatin, Cetuximab

## Abstract

**Background:**

The addition of cisplatin or cetuximab to radiotherapy in patients with locally advanced squamous cell carcinoma of the head and neck (SCCHN) has significantly improved the outcome. While the superiority of cisplatin over cetuximab in combination with radiotherapy has been shown in a definitive setting, we set out to compare postoperative chemoradiotherapy with cisplatin to radioimmunotherapy with cetuximab and radiotherapy alone within the Austrian head and neck cancer registry of the Working Group on Pharmaceutical Tumor Treatment (AGMT) study group.

**Material and methods:**

In the AGMT head and neck cancer registry, data of 557 patients with SCCHN from five Austrian cancer centers were prospectively collected between 2012 and 2017. Of these patients 120 received postoperative chemoradiotherapy with cisplatin, 26 patients received postoperative radioimmunotherapy with cetuximab and 56 patients were treated with adjuvant radiotherapy only. Patient characteristics, stage of disease, details on treatment as well as survival were analyzed by a chart-based review.

**Results:**

In patients treated with postoperative radiotherapy the addition of cisplatin significantly improved progression-free survival (PFS) and overall survival (OS) compared to cetuximab (PFS 84.2 months vs. 17.0 months, *p* = 0.04, OS not reached vs. 46.0 months, *p* = 0.02) and PFS compared to radiotherapy alone (PFS 84.2 months vs. 28.5 months, *p* < 0.01). Patients treated with cetuximab were significantly older and had a worse performance score than patients receiving cisplatin or radiotherapy alone.

**Conclusion:**

This study confirmed the importance of multimodal treatment concepts in patients with locally advanced SCCHN. Postoperative cetuximab might be an option in patients not eligible for high-dose cisplatin but cisplatin should remain the standard of care.

## Background

Squamous cell carcinoma of the head and neck (SCCHN) is the sixth most common cancer type worldwide and accounts for 1–2% of all cancer deaths [1]. Depending on the localization of the primary tumor, between one third and more than half of all patients are diagnosed with locally advanced disease. A curative treatment concept for these patients usually requires a multimodal approach by experienced head and neck surgeons, radiation oncologists and medical oncologists [2]. It was shown that the addition of high dose cisplatin to radiotherapy results in an overall survival (OS) benefit after resection of the primary tumor [3, 4] as well as in patients with unresectable disease [5].

Despite the fact that the incidence of human papilloma virus (HPV) positive oropharyngeal cancer is rising, the majority of cases of SCCHN in Europe are still associated with tobacco use and alcohol consumption and only 31% are with associated HPV [6, 7].

Due to the associated comorbidities caused by substance abuse, many patients outside clinical studies are not eligible for chemoradiotherapy (CRT) with cisplatin. In these patients, other radiosensitizing strategies including monoclonal antibodies, such as cetuximab, which has a more favorable safety profile than cisplatin, might be an attractive option. In fact, the superior efficacy of definitive radioimmunotherapy (RIT) with cetuximab compared to definitive radiotherapy (RT) alone was shown in a randomized phase III trial [8]; however, CRT with cisplatin has already been shown to be superior to RIT with cetuximab in a definitive setting through various prospective and retrospective studies [9–11]. In the postoperative setting, the monoclonal antibody has not yet been extensively studied or compared to adjuvant CRT with cisplatin or radiotherapy alone and is therefore not approved.

The head and neck cancer registry of the Working Group on Pharmaceutical Tumor Treatment (AGMT) prospectively collected the clinical data of patients with head and neck cancer treated at large Austrian cancer centers. The treatment strategies were based on investigators choice and some patients were treated with RIT with cetuximab in a postoperative setting. Within this analysis we set out to compare adjuvant chemoradiotherapy with cisplatin to radioimmunotherapy with cetuximab and radiotherapy alone in patients with locally advanced SCCHN in a real-world setting.

## Methods

All patients were prospectively included in the registry after providing written informed consent. The registry had passed the approval of the ethics committees at the participating institutions and the central ethics committee of the Province of Salzburg (415-E/1313). We collected the clinical characteristics and follow-up data of all included patients diagnosed with head and neck cancer at participating Austrian hospitals. For this analysis we selected all included patients who were treated with postoperative radiotherapy alone or in combination with cisplatin or cetuximab.

The median follow-up for all patients in our analysis was 60.9 months. Clinical data including the stage of disease (according to the 7th edition TNM staging system of the American Joint Committee on Cancer (AJCC) [12]), OS and progression-free survival (PFS) were analyzed by chart-based review. Furthermore, details on treatment strategies including surgery, radiotherapy as well as systemic therapies were documented via an electronic tool. The PFS was calculated from the date of primary diagnosis until disease progression or death from any cause and OS was defined as the time between primary diagnosis and death from any cause.

Statistical analyses were performed using IBM® SPSS® statistics software for Windows, version 24.0 (IBM Corp., Armonk, NY, USA). Where appropriate, Kruskal-Wallis test and Pearson’s χ^2^-test were used to compare the clinical characteristics of different treatment groups. For survival analyses, Kaplan-Meier curve analyses and the log-rank statistic were applied. A two-sided *p*-value of 0.05 was considered statistically significant. Factors with a significant influence on survival in univariate Cox regression analyses were used for multivariate analyses [13].

## Results

### Adjuvant treatment in patients with SCCHN

Among 218 patients treated with postoperative radiotherapy, 162 patients received treatment combined with systemic treatment, 120 of which received cisplatin and 26 received cetuximab (the remaining 16 patients were treated with RT in combination with carboplatin or mitomycin C), and 56 patients were treated with RT alone. Patients receiving RT or CRT were younger (median age 58.0 years for RT and RCTX vs. 71.5 years for RIT, *p* < 0.01) and had a better performance score (Eastern Cooperative Oncology Group [ECOG] > 1 in 8.9% RT vs. 1.7% CRT vs. 34.6% RIT of the patients, *p* < 0.01) compared to patients treated with cetuximab. Disease stages of patients receiving RT alone were more often lower (17.5% RT vs. 2.5% CRT vs. 3.8% RIT of patients with AJCC stage 1 or 2 disease, *p* < 0.01) than in the other groups. Most patients in the CRT and RIT groups received intensity-modulated radiation therapy (56.7% RCTX vs. 76.9% RIT vs. 31.6% RT, *p* < 0.01), while more patients received conventional 3D radiotherapy in the RT group. Apart from that there were no differences in clinical and treatment characteristics between the three groups. Radiotherapy was stopped early in 1.7% of the patients treated with cisplatin, 3.8% of the patients receiving cetuximab and 1.8% of patients treated with radiotherapy alone (*p* = 0.79) and most patients receiving CRT and RIT also completed the planned systemic therapy (77.5% in the cisplatin group and 76.9% in the cetuximab group, *p* = 0.95) (Table 1).

**Table 1:** Characteristics of patients treated with postoperative CRT, RIT and RT

	CRT	RIT	RT	*p*-value
	(*n* = 120)	(*n* = 26)	(*n* = 56)	
*Age (years)*				*<0.01*^*a*^
Median	58.0	71.5	58.5	
Range	33.0–75.0	56.0–89.0	43.0–91.0	
*Sex (%)*				*0.55*^*b*^
Male	79.2	88.5	80.4	
Female	20.8	11.5	19.6	
*AJCC stage (%)*				*<0.01*^*b*^
1	0.8	0.0	7.1	
2	1.7	3.8	10.7	
3	21.7	11.5	28.6	
4	72.5	69.2	42.9	
*ECOG score (%)*				*<0.01*^*b*^
0–1	89.2	65.4	59.0	
2–4	1.7	34.6	8.9	
*Resection margin (%)*				*0.38*^*b*^
Negative	52.5	50.0	53.6	
Positive	28.3	34.6	17.9	
*IMRT (%)*				*<0.01*^*b*^
Yes	56.7	76.9	32.1	
No	36.7	19.2	51.8	
*Radiotherapy discontinuation (%)*				*0.79*^*b*^
Yes	1.7	3.8	1.8	
No	95.0	96.2	91.1	
*Systemic therapy discontinuation (%)*				*0.95*^*b*^
Yes	22.5	23.1	–	
No	77.5	76.9	–	

Some values do not add up to 100% due to missing data

*CRT* chemoradiotherapy with cisplatin, *RIT* radioimmunotherapy with cetuximab, *RT* radiotherapy, *AJCC* American Joint Committee on Cancer, *IMRT* intensity modulated radiotherapy, *ECOG* Eastern Cooperative Oncology Group

^a^Kruskal-Wallis test

^b^Pearson’s χ^2^-test

**Fig. 1:**
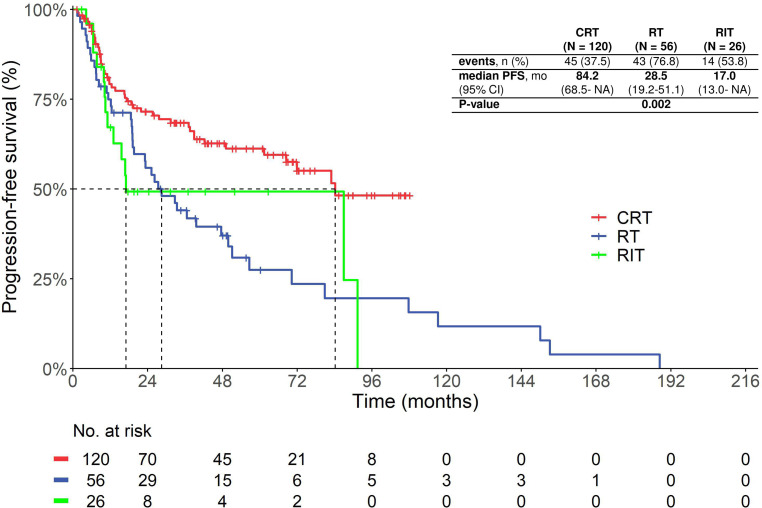
Kaplan-Meier curve showing the progression-free survival of patients treated with postoperative chemoradiotherapy, radioimmunotherapy and radiotherapy. *CRT* radiochemoradiotherapy with cisplatin, *RIT* radioimmunotherapy with cetuximab, *RT* radiotherapy

The median PFS of patients treated with postoperative CRT was 84.2 months (no 95% confidence interval, CI), while patients treated with RIT had a median PFS of 17.0 months (95% CI 0.0–60.3 months) and the patients receiving only RT had a median PFS of 28.5 months (95% CI 17.1–39.4 months, overall *p* < 0.01; Fig. 1). When compared to each other, the CRT group had a significantly longer PFS than the RT group (hazard ratio, HR 0.48, 95% CI 0.31–0.75; *p* < 0.01) and the RIT group (HR 0.54, 95% CI 0.29–0.98; *p* = 0.04), while the PFS of the RT group was not significantly longer than that of the RIT group (HR 0.98, 95% CI 0.53–1.82; *p* = 0.96).

**Fig. 2:**
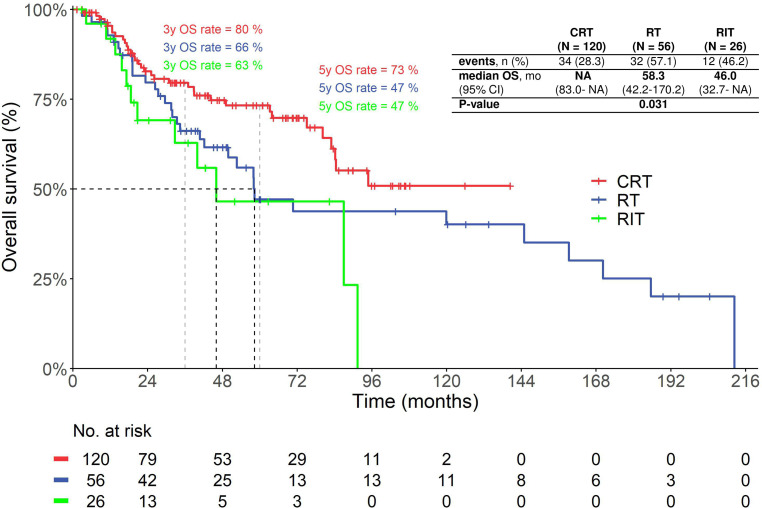
Kaplan-Meier curve showing the overall survival of patients treated with postoperative chemoradiotherapy, radioimmunotherapy and radiotherapy. *OS* overall survival, *CRT* radiochemoradiotherapy with cisplatin, *RIT* radioimmunotherapy with cetuximab, *RT* radiotherapy

The 3‑year and 5‑year survival rates of patients treated with postoperative CRT were 79.6% and 73.3%, respectively, while they were only 62.9% and 46.6% in the RIT group and 66.1% and 47.1% in the RT group. The median OS of patients treated with postoperative RCTX was not reached (no 95% CI), while patients treated with RIT had a median OS of 46.0 months (95% CI: 9.5–82.5 months) and the patients receiving only RT had a median OS of 58.3 months (95% CI: 36.0–80.7 months, overall *p* = 0.03; Fig. 2). When compared to each other, the OS in the CRT group was significantly longer than in the RIT group (HR: 0.44, 95% CI: 0.22–0.85; *p* = 0.02), but not significantly longer than in the RT group (HR: 0.65, 95% CI: 0.39–1.07; *p* = 0.09). The RT group also had no significantly longer OS than the RIT group (HR: 0.68, 95% CI: 0.34–1.35; *p* = 0.26).

### Factors influencing survival in patients treated with adjuvant systemic therapy

Apart from the choice of systemic therapy given in addition to radiotherapy, other factors such as age, performance score, AJCC stage and resection margins had no statistically significant influence on PFS and OS of patients treated with postoperative combined modality treatment (Table 2 and 3). Most patients with recurring disease received further treatment (75.6% of patients in the cisplatin group and 71.4% of patients in the cetuximab group, *p* = 0.68). Second line treatment consisted of systemic therapy in 53.8% and localized treatment strategies in 46.2% of the patients with known values (88.6%).

**Table 2:** Prognostic factors on progression free survival in postoperative CRT and RIT

	Univariate		
	HR	95% CI	*p‑value*^*a*^
*ECOG*			
ECOG >1 vs. 0–1	2.11	0.90–4.97	*0.09*
*Drug regimen*			
Cetuximab vs. cisplatin	1.37	1.01–1.85	*0.04*
*Age (years)*			
Age >60 vs. age <60	1.25	0.75–2.08	*0.40*
*R‑status*			
Rx, R1, R2 vs. R0	1.38	0.78–2.42	*0.28*
*AJCC stage*			
Stage 4 vs. stage 3	1.48	0.76–2.89	*0.25*

*CRT* chemoradiotherapy with cisplatin, *RIT* radioimmunotherapy with cetuximab, *HR* hazard ratio, *CI* confidence interval, *AJCC* American Joint Committee on Cancer, *ECOG* Eastern Cooperative Oncology Group

^a^Cox regression analysis

**Table 3:** Prognostic factors on overall survival in postoperative CRT and RIT

	Univariate		
	HR	95% CI	*p‑value*^*a*^
*ECOG*			
ECOG >1 vs. 0–1	1.76	0.62–5.00	*0.29*
*Drug regimen*			
Cetuximab vs. cisplatin	1.51	1.09–2.11	*0.02*
*Age (years)*			
Age >60 vs. age <60	1.52	0.84–2.73	*0.16*
*R‑status*			
Rx, R1, R2 vs. R0	1.42	0.76–2.63	*0.29*
*AJCC stage*			
Stage 4 vs. stage 3	1.26	0.61–2.56	*0.53*

*CRT* chemoradiotherapy with cisplatin, *RIT* radioimmunotherapy with cetuximab, *HR* hazard ratio, *CI* confidence interval, *AJCC* American Joint Committee on Cancer, *ECOG* Eastern Cooperative Oncology Group

^a^Cox regression analysis

## Discussion

Significant therapeutic challenges in daily practice arise from the fact that patients with locally advanced SCCHN are frequently symptomatic and often suffer from relevant comorbidities. Therefore, treatment in real-world populations may differ from protocols tested in selected cohorts in randomized trials [3, 4, 8, 14]. The AGMT head and neck cancer registry did not give recommendations on preferred treatment options but aimed at prospectively documenting current treatment strategies for advanced SCCHN in different tertiary cancer centers around the country. Thus, we report an unselected cohort of patients with distribution of age, primary tumor site and stage similar to recent literature [11, 15]. The majority of patients within the registry were treated with postoperative or definitive CRT or RIT.

Cetuximab is not approved in the postoperative setting of locally advanced SCCHN, as no study has been published comparing postoperative RIT to radiotherapy alone or CRT. One study (NCT00956007) that randomized patients between postoperative radiotherapy alone vs. radiotherapy with cetuximab has finished recruiting but first results are still pending; however, the safety of postoperative cetuximab administration was shown in two trials where the monoclonal antibody was combined with chemoradiotherapy [16, 17]. Within the Austrian head and neck registry, 26 patients who were not eligible for CRT with cisplatin but had high AJCC stage or positive resection margins were treated by postoperative RIT with cetuximab. The rate of early radiotherapy discontinuation was low and comparable to those seen in postoperative RCTX with cisplatin. Survival was significantly better in patients treated with postoperative RCTX with cisplatin compared to postoperative RIT with cetuximab or RT alone. Patients receiving RIT with cetuximab also did not live longer than patients treated with RT alone; however, as patients receiving cetuximab were significantly older, had a worse performance score and more advanced stage diseases, the lack of differences in survival could perhaps indicate a compensation of known poor prognostic factors through the addition of cetuximab to RT; however, age, performance score, tumor stage and resection status did not significantly influence survival of patients treated with postoperative RCTX with cisplatin or RIT with cetuximab. Data about the rate of extranodal extension, which has been shown as a negative prognostic factors, were not collected because staging was done in analogy to the 7th edition TNM Classification for Head and Neck cancer (extranodal extension was only added to the 8th edition in 2017 [18]) during time of recruiting. Certainly, these data have to be interpreted cautiously as the number of patients who received RIT with cetuximab was low and patients receiving CRT with cisplatin or RT alone were younger and had better performance scores.

Our work has some limitations. Even though prospective, center-based registries might be a better representation of real-world populations compared to clinical trials, some kind of selection bias cannot be excluded. It also has to be noted that while the AGMT head and neck registry is a prospectively maintained database, this analysis was done retrospectively. Furthermore, treatment was not randomized or followed uniform guidelines but was chosen at the discretion of the treating physicians. In the postoperative cohort treated with CRT, RIT or RT, the subgroups from the different treatment centers were too small to draw relevant conclusions concerning differences in patient survival between centers. Although it was shown that patients treated with RIT were older and had worse performance scores, more detailed information on specific comorbidities or risk factors driving treatment decisions in clinical practice would have been interesting.

In summary, our work confirms the importance of multimodal treatment concepts in patients with locally advanced SCCHN. The data of the AGMT head and neck cancer registry show that the addition of cetuximab to postoperative radiotherapy is well tolerable and might be an option for selected patients who are not eligible for high dose cisplatin; however, group size was small, and no survival benefit could be shown.
